# Investigations of Phytoconstituents, Antioxidant and Anti-Liver Cancer Activities of *Saueda monoica Forssk* Extracted by Microwave-Assisted Extraction

**DOI:** 10.31557/APJCP.2020.21.8.2349

**Published:** 2020-08

**Authors:** Ali A. A. Al-Shawi, Mustafa Fadhil Hameed, Nashwan Hussein Ali, Kawkab Ali Hussein

**Affiliations:** 1 *Department of Chemistry, College of Education for Pure Sciences, University of Basrah, Basrah, Iraq. *; 2 *Ministry of Education, General Directorate of Education in Basrah, Basrah, Iraq. *; 3 *Department of Applied Chemistry, College of Applied Sciences, University of Samarra, Iraq. *

**Keywords:** Apoptosis, cell cycle, liver cancer, natural antioxidants, Saueda monoica Forssk

## Abstract

**Background:**

Wild edible plants are good sources for bioactive compounds, vitamins, and minerals with various applications. They can play a role in supporting the immune system and are highly beneficial as resources. Suaeda monoica Forssk is a wild edible plant that grows in Iraq and it’s biological activities have not yet reported.

**Methods:**

*Saueda monoica Forssk* bioactive compounds were extracted by a microwave-assisted extraction method using ethanol as a solvent, and its chemical composition was analyzed by GC-MS. The biological activities were evaluated via antioxidant, anti-liver-cancer, antibacterial, and toxicity tests in vitro.

**Results:**

The results of GC-MS analysis showed that there were about 20 bioactive compounds. The most abundant compound was N,N-Dimethylglycine methyl ester, followed by 9,12,15-Octadecatrienoic acid, n-Hexadecanoic acid, and N,N-Dimethylglycine. The antioxidant activity of the ethanol extract of the plant showed a significant IC_50_. The extract of *S. monoica* against liver cancer cells (HCAM) showed significant toxicity. Flow cytometric analysis showed a significant induced apoptosis and cell cycle arrested at G1 phase.

**Conclusions:**

The results indicated the significance of the components of Iraqi S. monoica Forssk by MAE method as a potential food supplement in nutrition systems to prevent liver cancer and enhance the liver’s defense against diseases.

## Introduction

Wild edible plants are an important source of edible healthy food due to the nature of the chemical components in the fruits, leaves, roots, tubers, and rhizomes, which could have applications in medical uses and daily diet (Bharucha et al., 2010). Common wild edible plants include amaranth, asparagus, burdock, cattail, clovers, chickweed, and chicory (Tariku and Eyayo, 2017). They are reported to be used as ointments for wounds and possess antiviral activity. Various countries have been using wild edible plants species, like India, China, and some Arab and African countries (Geng et al., 2016). A number of wild edible plants grow in Iraq, but few studies have reported on their chemical compositions. One report showed the bacterial activity of 22 Iraqi wild plants (Abdulameer, 2014). One of the most famous wild edible plants in Iraq is *Suaeda monoica Forssk*, which belongs to the family *Chenopodiaceae* and grows in hypersaline soil (Iwona et al., 2014). It is used by Iraqi people in the southern region as a vegetable food and is available in vegetable shops or markets. It is believed to have health benefits, it has a good taste, and is non-toxic (Ali et al., 2018). One study investigated the phytoconstituents of a methanol extract of the wild plant in Saudi Arabia (Elsharabasy et al., 2019). People are suffering from liver diseases due to viruses, drugs, poisons, alcohol, cancer, and inherited diseases (Xiong and Guan, 2017). The long tradition of medicinal plants that have been used liver disease treatment could help to discover new plants with new bio-functions to prevent liver diseases and understand the mechanisms of action (Li et al., 2018). Wild plants could play a role in hepatic protection due their antioxidant content, which could prevent damage from reactive oxygen species (ROS) and inhibit free radical generation (Guan and He, 2015). The phyto-nutrients discovered in wild plants are growing and can be explored using different extraction methods and solvents (Traka and Mithen, 2011). Saline soils and environmental factors play a role in the chemical composition of wild plants (Ceccanti et al., 2019). In addition, their toxicity has to be evaluated, and the compositions of toxic compounds have to be determined to identify the toxicity of edible species and the safety of high and low-toxicity compounds like nitrites and oxalate (Yang et al., 2020). Thus, examining the toxicity of wild edible plants is very important to demonstrate the limits or high benefits of the components, while taking into consideration the development of cultivation techniques, which can also control or reduce the toxic compounds in the plants (Cornanra et al., 2018). Hence, Few reports have been used microwave-assisted extraction (MAE) for wild plants components with various solvents and compared with other extraction methods such as maceration and soxhlet extraction methods (Li et al., 2017). MAE exhibited high selective efficiency for the bioactive compounds (Belwal et al., 2017). The biological properties of wild plants components extracted by MAE have not yet reported, and one report showed high antioxidant activity of three wild edible mushrooms extracted by MAE (Özyürek et al., 2014). Therefore, in the present research, used microwave-assisted extraction and ethanol solvent to investigate the bioactive compounds, antioxidant, antibacterial, and anti-liver cancer properties of *S. monoica Forssk* (wild edible plant).

## Materials and Methods

**Figure 1:**
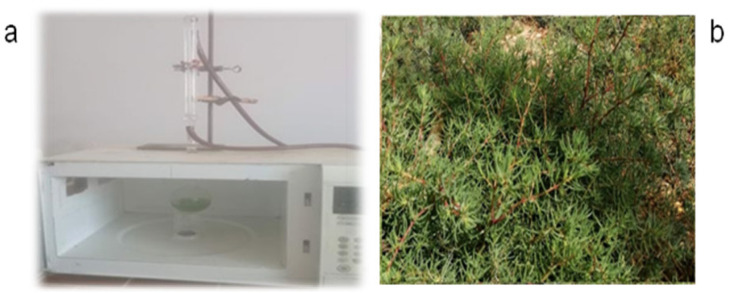
a) Microwave-assisted extraction (homemade). b) Iraqi S.* monoica Forssk* wild edible plant

*Saueda monoica Forssk* wild edible plant was collected from a farm in the southern area in Basrah province, Al-Seebah district, southern of Iraq on February, 2019, as shown in (Figure 1 (b)). The wild plant was cleaned, sliced, dried under sunlight exposure for several days, ground mechanically and kept at 4 ºC for further working. Microwave-assisted extraction (MAE) homemade connected with cooling bath and ethanol solvent 99% as extract solvent. The study period conducted from April, 2019 to December, 2019. All the reagents purchased from sigma Aldrich, BDH, UK and USA biological.

*Methods*

*monoica Forssk extraction method by MAE*

In a round conical flask added 5 g of *monoica Forssk* powder with 100 ml of absolute ethanol 99% and mixed well then left for 20-30 min inside microwave-assisted extraction homemade instrument (Daewoo company, S. Korea), and started the extraction process under conditions of 1 min and 50 ºC, as shown in (Figure 1(a)). The ethanol extract was cooled for 10-20 min and centrifuged with 15 min and 3000 rbm and then filtered by whatman filter paper 20 µm and kept at 4 ºC. 

*GC-MS analysis method of S. monoica Forssk ethanol extract by MAE*

Gas chromatography mass spectrometric analysis was performed employing a GC coupled to Mass Hunter workstation software (Agilent 7890B GC with 5977A MSD, USA). Phenyl methyl siloxane %5 column, under pressure 6.0799 psi, the column temperature gradient was initiated at 40°C and a linear gradient was obtained by raising the temperature from 50 to 280 °C at the rate of 10°C/min. The injector was maintained at 290°C and 4 min solvent stopped. Helium was used as the carrier gas at a flow rate of 1 ml/min. Type of injection: pulsed splitless, molecular weight test range 35-650 m/z, test rate 1562 (N2). The extracts were filtered through syringe filters and 1 μl of ethanol extract was injected into the GC column and analyzed according to NIST library.

*Antioxidant activity method*

*S*. *monoica Forssk* (MF) ethanol extract was used to evaluate antioxidant activity using DPPH (2,2-Diphenyl-1-picrylhydrazyl), according to Young and his group method with some modification (Lee et al., 2013). Briefly, various concentrations of MF (10,20,30,40,50,60,70, 80) µl were added to 200 µl of DPPH (1mm) in 96 well/plate and kept in a dark place for 30 min, and measured the absorbance at 490 nm by a micro-plate reader (ELISA, Asyshitech., UK), the equation (1) was used to estimate antioxidant activity % of MF:

Antioxidant activity % = {1- [ A_s_/ A_c_]} x 100 1

Where: A_s_ = absorbance of *monoica Forssk* +DPPH ; A_c_ = absorbance of DPPH as control

*Anticancer activity*

*Cell culture*

Liver cancer HCAM cells (Provided by Iraqi national center for cancer researches, University of Al-Mustansriah, Baghdad, Iraq) were maintained in 10 cm plate contained RPMI-1640 upplemented with 10% FBS, 100 units/ml penicillin and 100 μg/ml streptomycin at 37°C in a humidified atmosphere with 5% CO2.

*MTT assay*

MTT assay was used to determine IC_50_ values of *S. monoica Forssk* on the viability of liver cancer cells HCAM as described in with some modifications (Ali et al., 2011). Briefly, liver cancer cells were plated at a density of 1x104 cells per well in 96-well plates. After 24 h, cells were treated with 100 μl of complete culture medium containing (400, 800, 1000, 1200, 1400 and 1800) µg/ml of plant extract with an equal amount of DMSO as negative control. After incubation for 24 h, cell viability was determined, 10 μl of MTT (5 mg/ml) in phosphate buffered saline was added to each well and incubated for 4 h. After removal of the medium, 150 μl DMSO was added to each well and shaken carefully, the absorbance was recorded by micro-plate reader (ELISA, Asyshitech., UK) at a wavelength of 620 nm, the inhibition ratio of compounds on cell growth was calculated using equation (2) (Al-Shammari et al., 2016):

Cell viability % = {[A620 (control) – A620 (treated)] / A620 (control)} x 100 2

Acridine orange / Ethidium bromide (AO/EB) staining

According to Arumugam and his group method (Arumugam et al., 2019), Trypsin fersin was added to HCAM cells by trypsinization process and then added medium RPMI-1640, put a clean and sterilized slide on the planted cells dish and start planted 5,000 of HCAM cells on the slide cover and then covered tightly it by parafilm sheet for 24 h in the incubator 5% CO_2_ and 37 ºC. After 24 h, discard the medium and added IC_50_ value of MF, then close the dish tightly and re-incubate for 24 h, next, raised up the slide cover and put it in a clean slide and added 70 µl of AO/EB stain, and immediately take the slide under Fluorescent microscope (Flourecent Microscope, Zeiss axiolabe, CE, Germany) to record the photos.

*Flow Cytometric Analysis of apoptosis*

Flow cytometric used to identity of late apoptosis according to Mohammad and his group method with some modification (Khan et al., 2012). Briefly, HCAM cells were treated with or without IC_50_ values of *monoica Forssk* extract for 24 h. The cells were harvested, rinsed twice with PBS, and labeled with 5 uL FITC-conjugated annexin V according to the manufacturer’s instructions. After incubation in dark for 10 min and then labeled with PI, the samples were immediately analyzed on a flow cytometer (Beckman Coulter, Epics XL).

*Flow Cytometric Analysis of Cell Cycle*

Flow cytometric used to identify G1, S and G2 gates in the cell cycle of liver cancer cells. According to Mohammad and his group method with some modification (Khan et al., 2012). HCAM cells were treated with or without IC_50_ values of *monoica Forssk* extract for 24 h. The cells were then washed with PBS and fixed with 70% ice-cold ethanol at 4◦C for overnight. After washing twice with PBS, cells were stained with a solution containing 50 μg/mL of PI and 100 μg/mL RNase A for 30 min in the dark at room temperature. The stained cells were analyzed by flow cytometry (Beckman Coulter, Epics XL).

*Statics analysis*

The experiments of DPPH and MTT methods were repeated in 4 wells. The data was analyzed by GraphPad Prism 8.1. *P < 0.0001 were considered as statistical significant.

## Results

*GC-MS analysis of S. monoica Forssk ethanol extracted by MAE*

GC-MS is a suitable method to analysis the chemical constitute for medicinal herbs extract. Control the conditions will clarify the components. Therefore, in this research used GC-MS method to analysis chemical components of *S.* *monoica Forssk* ethanol extracted by MAE. the analysis results revealed about 20 bioactive compounds, as shown in Table 1. The highest peak was detected for compound 2 (Glycine, N,N-Dimethyl-, methyl ester, RT= 6.415 min), followed by compound 11 ( 9,12,15-Octadecatrienoic acid, RT= 24.085 min), compound 9 (n-Hexadecanoic acid, RT= 22.438 min), compound 4 ( N,N-Dimethylglycine, RT=14.906 min), and others according to the NIST library. 

*Antioxidant activity by DPPH method*

**Figure 2:**
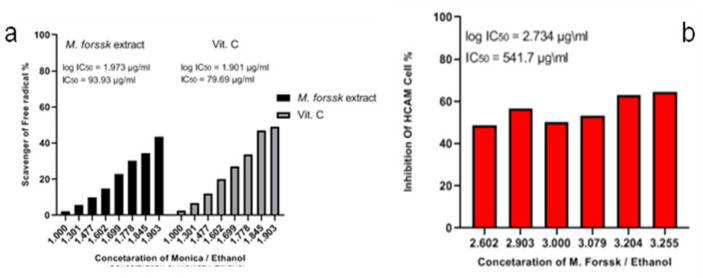
a) Antioxidant activity of * monoica Forssk* ethanol extract using DPPH method, and vitamin C as positive control; b) MTT assay of * monoica Forssk* ethanol extract against HCAM liver cancer cells (showed moderate action with IC_50_ value= 541.7 µg/ml)

DPPH assay is a simple method for evaluating the scavenger activity of medicinal herb extracts. Here, used micro-plate reader (96 plate/well) with different concentrations of monoica extract (10-80) µl and mixed with a constant concentration of DPPH and kept in a dark place for 20-30 min. Color changed to transparent indicated the scavenger ability of extract against DPPH. The results showed significant antioxidant action: the color of the test solution changed from dark violet to transparent after storing it for 30 min. The IC_50_ value was 93.93 µg/ml, which was compared with IC_50_ value of vitamin C (79.69 µg/ml), as shown in (Figure 2 (a)).

*Anti-liver cancer properties of S. monoica Forssk ethanol extracted by MAE*

*MTT assay and AO/EB methods*

**Figure 3:**
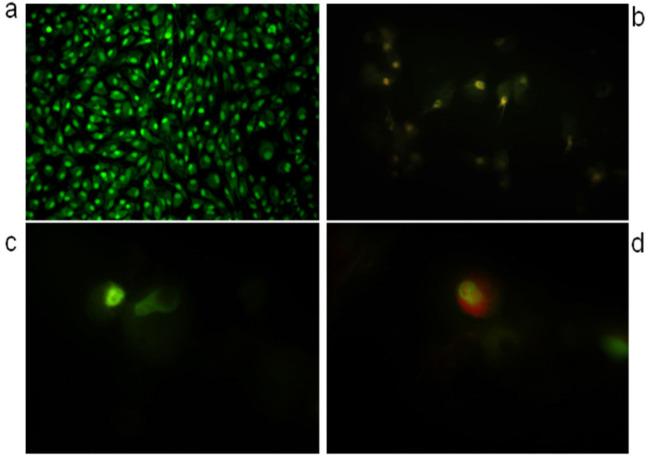
AO/EB Staining of * monoica Forssk* Extract to Detect Apoptosis and DNA Damaged: a. Untreated cells; b, Cells treated with * monoica Forssk* extract; c and d, Cells treated with *monoica* Forssk ethanol extract

**Figure 4:**
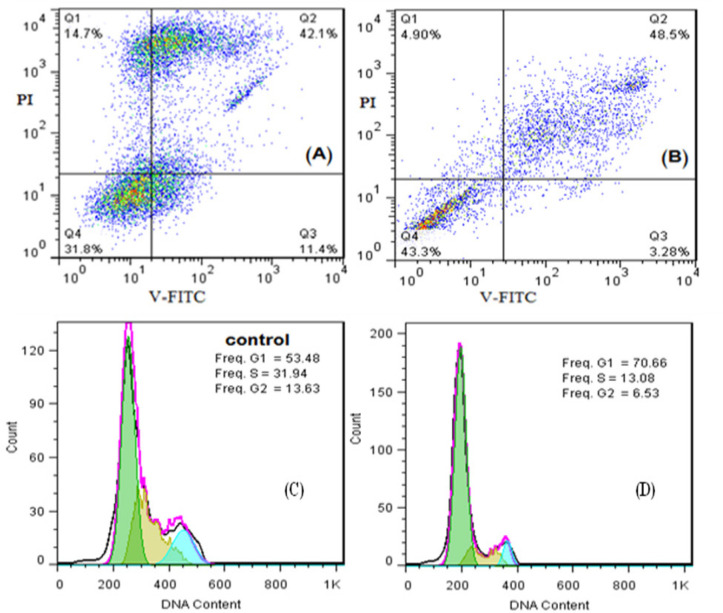
Flowcytometric Analysis of IC_50_ Value of *monoica Forssk* Extract Against HCAM Cancer Cells Showed: A, Cells treated with extract (Live cells (Q4)= 31.8, early apoptosis (Q1)= 14.7 and late apoptosis (Q2)= 42.1); B, Untreated cells (Live cells (Q4)= 43.3, early apoptosis (Q1)= 4.9 and late apoptosis (Q2)= 48.5); C, Cell cycle arrest of cells treated with extract; D, Cell cycle arrest of untreated cells

The prescreening of monoica extract showed an inhibition of growth cells ratio (40-50%) against liver cancer cells (HCAM) by MTT assay. MTT assay used to estimation of IC_50_ for 24 h. GraphPad prism software used to estimate IC_50_ and was equal to 541.7 µg/ml. Furthermore, AO/EB fluorescent staining used to detect apoptosis form in the morphological changes of treated and untreated HCAM cells, as showed in as shown in (Figure 3). AO/EB method supportd by flowcytometric analysis method for estimating early apoptosis, late apoptosis, necrosis, and live cells ratios of IC_50_ value of monoics extract. The results of flowcytometric analysis showed that value of early apoptosis (Q1=4.9%), late apoptosis (Q2=48.5%), necrosis (Q3=3.28%) and live cells (Q4=43.3%) compared with the control values early apoptosis (Q1=14.7%), late apoptosis (Q2=19.4%), necrosis (Q3=11.4%) and live cells (Q4=54.5%). Likewise, flowcytometric analysis used to estimate and detect the phases (G1, S, G2) in cell cycle, as shown in (Figure 4 (A,B,C,D)). S and G2 phases of cell cycle for HCAM cells treated with monoica extract showed values lower than the control, while G1 in the treated cells higher than control. 

**Table 1:** Showed the GC-MS Chemical Compositions Analysis of *monoica* Forssk Ethanol Extract by MAE (Showed 8 Bioactive Compounds with High Peaks, Indicated with Red Color)

No.	RT in min	Name of compound	Molecular Formula
1	4.434	1-Propanone, 1-(1-adamantyl)-3-dimethylamino-	C_15_H_25_NO
2	6.415	Glycine, N,N-dimethyl-, methyl ester	C_15_H_25_NO
3	7.999	Glycine, N,N-dimethyl-, ethyl ester	C_6_H_13_NO_2_
4	14.906	N,N-Dimethylglycine	C_4_H_9_NO_2_
5	17.414	2H-Benzo[f]oxireno[2,3-E]benzofuran-8(9H)-one, 9-[[[2-(dimethylamino)ethyl]amino]methyl]octahydro-2,5a-dimethyl-	C_19_H_32_N_2_O_3_
6	19.728	Pterin-6-carboxylic acid	C_7_H_5_N_5_O_3_
7	21.153	Phytol, acetate	C_22_H_42_O_2_
8	21.591	1,2-15,16-Diepoxyhexadecane	C_16_H_30_O_2_
9	22.438	n-Hexadecanoic acid	C_16_H_32_O_2_
10	23.786	Phytol	C_20_H_40_O
11	24.085	9,12,15-Octadecatrienoic acid	C_18_H_30_O_2_
12	24.266	Octadecanoic acid	C_18_H_36_O_2_
13	24.391	Hexadecanamide	C_16_H_33_NO
14	25.913	9-Octadecenamide, (Z)-	C_18_H_35_NO
15	26.114	Phenol, 2,2'-methylenebis[6-(1,1-dimethylethyl)-4-methyl	C_23_H_32_O_2_
16	26.392	5,8,11,14-Eicosatetraenoic acid, methyl ester	C_21_H_34_O_2_
17	27.073	Hexadecanoic acid, 2-hydroxy-1-(hydroxymethyl)ethyl ester	C_19_H_38_O_4_
18	29.991	n-Tetracosanol-1	C_24_H_50_O
19	34.133	Stigmasterol	C_29_H_48_O
20	35.057	gamma.-Sitosterol	C_29_H_50_O

## Discussion

The studies have shown that microwave-assisted extraction is an important method for the extraction of bioactive compounds because it is selective, fast, and economical, and it may replace other extraction methods to discover new natural compounds. The chemical composition of *S.* *monoica Forssk* extracted by microwave-assisted extraction have not yet reported. Therefore, used an ethanol extract of *S.* *monoica Forssk* prepared via MAE method in constant conditions for the first time, as shown in Figure 1 (a). The compounds contain N and O in their structures as derivatives of amino acids, fatty acids, and phenol or toxic compounds such nitrites and oxalate. These are important for human health due to their biological functions and cannot be produced by the body. Compared the results to those of Elsharabasy and his group (Elsharaby et al., 2019), who used different solvents and extraction methods for wild Saudi Arabian *S. monoica Forssk*. The results exhibited some similar and selective bioactive compounds. The environmental effects of the soil play an important role in the secondary metabolism of wild plant growth to produce bioactive compounds, and recommend using microwave-assisted extraction as an extraction method in future experiments for the isolation of natural molecules from medicinal plants. 

The antioxidant actions of wild plants as food are important due to their functions in the body. Therefore, discovering new edible wild plants with high or moderate antioxidant activity could provide enhanced health benefits against human diseases. Carine and her group found that roots of *O. japonica *and fronds of *M. orientalis *were showed high antioxidant among all fern extracts (Carine et al., 2015). Hereby, used the DPPH method as a fast and inexpensive method with precise results to determine the antioxidant activity of the extract. The antioxidant action enhances the importance of *S*. *monoica Forssk* extracted by microwave-assisted extraction, which could possibly inhibit free radical formation in the body and prevent liver disease. Non of previous studies showed the antioxidant activity of monoica Forssk extracts and It’s the first report of antioxidant evaluation of Iraqi *monoica Forssk* ethanol extracted by MAE method.

The studies have been showed the importance of wild edible plants against cancer diseases. Jianling and his group found that C. sanguine extract showed significant inhibition against Hep-2 and MGC-803 tumor cells (Jiangling et al., 2013). Yiftach and his group found that Silybum marianum L. is antioxidant wild edible and enhance liver cells regeneration (Yiftach et al., 2008). Gnocchi and his group found that ethyl acetate of *Brassica oleracea *L. and *Crithmum maritimum L*. was promised in prevent growth of Hepatocellular carcinoma (Gnocchi et al., 2020). Mariangela and her group found that *Origanum vulgare L*. showed significant anti-proliferative against liver cancer cells HepG2 (Mariangel et al., 2015). Nasir and his group used percolation extraction method by ethanol and then used four solvent (dicholormethane, ethyl acetate,, hexane and n-butanol) for extract fractions of S. monoica. Among the four solvents, dichloromethane was the most active in cells proliferation of HepG2 and it subjected for isolation the four compounds from Saudi Arabic *Saueda monoica Forssk* and found among them only two compounds showed a potential action against liver cancer cells HepG2 (Nasir et al., 2020). 

The previous studies of Iraqi *S. monoica Forssk *extracted by MAE did not reported anti-liver or anticancer properties in Iraq and worldwide. In this research, the anti-liver cancer activity of Iraqi *S. monoica Forssk *extracted by MAE and ethanol as extraction solvent was investigated using an MTT assay. The results showed a significant toxicity against HCAM cancer cells, and the IC_50_ value was 541.7 µg/ml. This value indicates a substantial potency of the bioactive compounds. To enhance the MTT assay results, AO/EB staining method was used to detect apoptosis and DNA damage by using the IC_50_ value of the extract in comparison with control cells (untreated cells). Apoptosis phase occurred in the HCAM cells because of the bioactive compounds function together of monoica extract, which supported the MTT assay results. MTT result combined with Nasir and his group results of Saudian monoica as anti- liver cancer cells promised the future uses of this wild herb in the traditional medicine and prevent liver diseases. Therefore, urgent further investigations of used MAE to isolate natural molecules from Iraqi *S. monoica Forssk *ethanol extract by MAE which responsible for the anti-liver cancer in the near future.

To our knowledge, it’s a first report of apoptosis and cell cycle of S. monoica extracted by MAE against liver cancer cells HCAM. IC_50_ value of *monoica Forssk* used to detect early and late apoptosis of HCAM cancer cells for 24h. The results showed late apoptosis (Q2= 48.5%,) higher than the control value of untreated cells (Q2= 42.1%,) and reduced early apoptosis value (Q1). Maybe the time of extract exposure could play a function in increasing of late apoptosis ratio in time independent. In addition, G1, S, G2 phases of HCAM cancer cells cycle identified via IC_50_ value of *monoica Forssk* extract. The results showed reduced of G2 and S ratios, while increased G1 ratio in the treated cells compared with untreated cells. Indicated that *monoica Forssk* ethanol extracted by MAE arrested liver cancer cells cycle at G1 phase.

In conclusion the type of extraction method and solvent play a role in the selectivity of chemical compositions of wild edible plants or medicinal plants. The microwave-assisted extraction method with GC-MS analysis provided an initial library of bioactive compounds of *S. monoica Forssk* ethanol extract. The antioxidant and anti-liver cancer results could pave the way for the isolation of the molecules responsible for the anticancer action against HCAM liver cancer cells and mechanistic investigations. Therefore, suggested using regular daily doses of *S. monoica Forssk* to enhance the prevention of liver cancer and diseases.
